# Preconception to postpartum accelerometry-based 24-hour movement behaviors: a prospective cohort study

**DOI:** 10.1186/s12889-025-26034-4

**Published:** 2026-01-07

**Authors:** Anne H. Y. Chu, Natarajan Padmapriya, Shuen Lin Tan, Claire Marie J. L. Goh, Yap-Seng Chong, Lynette P. Shek, Kok Hian Tan, Peter D. Gluckman, Fabian K. P. Yap, Yung Seng Lee, See Ling Loy, Jerry K. Y. Chan, Keith M. Godfrey, Johan G. Eriksson, Shiao-Yng Chan, Jonathan Y. Bernard, Falk Müller-Riemenschneider

**Affiliations:** 1 Saw Swee Hock School of Public Health, National University of Singapore, Singapore, 12 Science Drive 2, Singapore, 117549 Singapore; 2 Institute for Human Development and Potential, Agency for Science, Technology and Research (A∗STAR), Singapore, Singapore; 3 Department of Obstetrics & Gynaecology, Yong Loo Lin School of Medicine, National University of Singapore, Singapore, Singapore; 4 Department of Paediatrics, Yong Loo Lin School of Medicine, National University of Singapore, Singapore, Singapore; 5 Department of Maternal Fetal Medicine, KK Women’s and Children’s Hospital, Singapore, Singapore; 6 Liggins Institute, University of Auckland, Auckland, New Zealand; 7 Duke-NUS Medical School, Singapore, Singapore; 8 Department of Pediatrics, KK Women’s and Children’s Hospital, Singapore, Singapore; 9 Department of Reproductive Medicine, KK Women’s and Children’s Hospital, Singapore, Singapore; 10 MRC Lifecourse Epidemiology Centre and NIHR Southampton Biomedical Research Centre, University of Southampton and University Hospital Southampton NHS Foundation Trust, Southampton, UK; 11 Department of General Practice and Primary Health Care, University of Helsinki, Helsinki, Finland; 12 Folkhälsan Research Center, Helsinki, Finland; 13 Université Paris Cité and Université Sorbonne Paris Nord, Inserm, INRAE, Centre for Research in Epidemiology and StatisticS (CRESS), Paris, France; 14 Berlin Institute of Health, Charite University Medical Centre, Berlin, Germany

**Keywords:** Preconception care, Pregnancy, Postpartum period, Accelerometry, Physical activity, Sedentary behaviors, Sleep, Movement, Cohort studies, Behavioral monitoring, Maternal health, Activity measurement, Longitudinal studies

## Abstract

**Background:**

Changes in movement behaviors – physical activity (PA), sedentary behavior, and sleep patterns – across preconception, pregnancy, and postpartum are associated with maternal and child health but remain understudied. Longitudinal accelerometer-measured data, including weekday-weekend differences, are lacking. Understanding these patterns is essential for developing targeted interventions that account for lifestyle variations. We investigated longitudinal changes in PA, sedentary behavior, and sleep patterns throughout preconception, pregnancy, and postpartum using prospectively collected accelerometry data.

**Methods:**

In a Singapore prospective preconception cohort, women aged 18–45 wore an accelerometer on their non-dominant wrist for seven days during preconception (within one year of planned conception), mid-pregnancy (24–28 weeks), and 12-month postpartum. Valid data required measurements at all three or at least two consecutive timepoints (preconception-pregnancy or pregnancy-postpartum). Changes in PA (vigorous-, moderate-, and light-intensity), sedentary behavior, and sleep were analyzed using generalized estimating equations.

**Results:**

Among 139 women (mean age: 30.8 years), most were under/normal weight (61.9%), Chinese (83.5%), had undergraduate education (59.0%), were employed (88.5%), and nulliparous (65.5%). Moderate- and vigorous-intensity PA decreased from preconception to mid-pregnancy, with vigorous-intensity PA remaining low postpartum, while moderate-intensity PA rebounded (daily mean [95% confidence interval] vigorous: 4.1 [2.8–5.4)], 1.7 [0–4.2], and 1.8 [0–5.0] min/day; moderate: 88.2 [82.8–93.5], 68.7 [58.6–78.7], and 90.2 [77.7–102.7] min/day, respectively). Light-intensity PA remained consistent from preconception to mid-pregnancy but increased postpartum (301.5 [289.6–313.5], 298.3 [273.1–323.5], and 340.1 [305.9–374.5] min/day, respectively). Sedentary behavior rose mid-pregnancy but decreased postpartum (618.2 [603.4–633.1], 639.6 [607.6–671.5], and 597.1 [553.5–640.7] min/day, respectively). Sleep duration remained stable from preconception to mid-pregnancy until postpartum, when it decreased (428.9 [420.6–437.3], 432.2 [412.2–452.1], and 408.4 [387.2–429.6] min/day, respectively). Moderate-/vigorous-intensity PA showed no weekday/weekend differences (daily percentage range, moderate: 4.7–6.6%; vigorous: 0.1–0.3%). Women engaged in less light-intensity PA on weekdays during mid-pregnancy and postpartum (weekdays: 20.5–23.2% versus weekends: 21.3–24.8%). Weekends showed lower sedentary behavior (weekdays: 42.5–45.4% versus weekends: 38.5–42.1%) and longer sleep duration (weekdays: 27.8–29.3% versus weekends: 29.8–32.0%) across all timepoints.

**Conclusions:**

Sustained moderate- and vigorous-intensity PA from preconception through postpartum should be promoted, particularly vigorous-intensity PA recovery postpartum. Light-intensity PA, which increased postpartum, could be leveraged to reduce sedentary behavior, especially on weekdays. Given postpartum sleep decline, strategies to support maternal sleep, particularly on weekdays, are needed.

**Clinical trial information:**

ClinicalTrials.gov, NCT03531658 (registered May 22, 2018).

**Supplementary Information:**

The online version contains supplementary material available at 10.1186/s12889-025-26034-4.

## Introduction

As key components of 24-h movement behaviors, physical activity, sedentary behavior, and sleep play crucial roles in maintaining lifelong well-being [1, 2]. Each behavior is unique, inherently displaces time available for others, emphasizing the constrained and reciprocal nature of daily movement patterns. Pregnancy and postpartum phases represent important stages of transition in a woman’s life, characterized by substantial physiological and lifestyle changes [3, 4], which may influence how women allocate time across these behaviors. Despite the well-documented health benefits of physical activity, global inactivity rates remain high, particularly among women [5]. Regular physical activity is associated with improved health outcomes across the reproductive lifespan, including preconception (e.g., infertility, polycystic ovary syndrome, weight management), pregnancy (e.g., reduced risk gestational diabetes, preeclampsia), and postpartum (e.g., weight retention, mental health) [6–8]. Similarly, sedentary behavior and sleep during pregnancy are integral to maternal health, with prolonged sedentary time linked to excessive gestational weight gain and diabetes [9, 10], and poor sleep associated with adverse maternal and child health [11].

Most existing research relied on self-reported data across various life stages: preconception-pregnancy [10, 12–15], pregnancy-postpartum [16–21], preconception-postpartum [15, 22–24], offering contextual insights but lacking the granularity of accelerometer-measured data. No accelerometry studies have comprehensively examined temporal trends in physical activity, sedentary behavior, and sleep throughout the entire preconception-to-postpartum transition. A previous cohort study [23], although prospective, relied on self-reported data and found increased walking but decreased moderate-to-vigorous physical activity (MVPA) from preconception to pregnancy, alongside declines in both screen and total sedentary behavior postpartum.

Unlike self-reported data, accelerometry captures the full spectrum of movement behaviors – including physical activity at varying intensities, sedentary time, and sleep – across the entire day. This comprehensive approach enables the examination of temporal variations in 24-h movement behaviors, such as weekday-weekend differences. Previous studies have reported mixed findings on weekday-weekend differences in maternal movement behaviors [25–27], but these primarily focused on parental behaviors rather than prospectively tracking changes from preconception through pregnancy and postpartum. Understanding weekday-weekend differences provides insight into how structured schedules (e.g., work, childcare) on weekdays contrast with the flexibility of weekends, enhancing the relevance of tailored interventions. Yet, weekday-weekend comparisons across the preconception-to-postpartum transition have not been thoroughly investigated.

This study aimed to describe the prospective changes in maternal 24-h movement behaviors (vigorous-, moderate-, light-intensity physical activity, sedentary behavior, and sleep) across preconception, mid-pregnancy, and postpartum; and comparing weekday and weekend differences of these behaviors. We hypothesized that (1) vigorous- and moderate-intensity physical activity would decline from preconception to pregnancy and remain low postpartum, (2) light-intensity activity would remain stable during pregnancy but increase postpartum, (3) sedentary behavior would increase during pregnancy and decrease postpartum, and (4) sleep duration would decline during pregnancy and further decrease postpartum. Further, we hypothesized that weekdays would show lower physical activity (of any intensity), higher sedentary behavior, and shorter sleep duration compared to weekends.

## Methods

### Study design and participants

The Singapore Preconception Study of Long-Term Maternal and Child Outcomes (S-PRESTO) is a prospective cohort study designed to investigate the impact of factors including nutrition, lifestyle, and maternal mood before and during pregnancy on offspring's epigenetics and health outcomes [28]. Briefly, from February 2015 to October 2017, 1,032 women aged 18–45 were enrolled, comprising Chinese, Malay, Indian, or mixed ethnicities, who were aiming to conceive and deliver in Singapore. After three preconception visits, those not achieving pregnancy within 12 months were withdrawn from the study (*n* = 557). For those who conceived (*n* = 475) and remained in the study and delivered a singleton (*n* = 373), further assessments were performed during and after gestation. Exclusion criteria included women who had been trying to conceive for over 18 months before recruitment; those currently pregnant, those who had used specific contraceptives or undergone fertility treatments in the past month, and those with health conditions including diabetes or taking systemic steroids, anticonvulsants, or recent medication for HIV or Hepatitis B/C. Written informed consent was provided by participants. Ethical approval was obtained from the SingHealth Centralised Institutional Review Board (reference 2014/692/D). This study is registered at ClinicalTrials.gov, NCT03531658 (registered May 22, 2018).

### 24-h movement behaviors

Participants were asked to wear ActiGraph wGT3X-BT accelerometers (ActiGraph, Pensacola, FL) to record their 24-h movement behaviors for seven consecutive days and nights (sampling rate of 80 Hz, based on its optimal balance between data resolution and storage efficiency [29, 30]) on their non-dominant wrist using the provided adjustable strap. Assessments were conducted during the first preconception clinic visit, the fourth pregnancy clinic visit at 24–28 weeks’ gestation, and the 12-month postpartum visit. To be valid for analysis, accelerometer data must include ≥ 16 h/day of wear time on at least three monitoring days (including at least one weekend day), consistent with wrist-worn accelerometer protocols from the UK Biobank cohort [31]. This approach ensures sufficient data capture while minimizing participant burden. Although shorter wear time thresholds (≥ 10 h) are common [32], the 16-h wear time threshold was applied to optimize data reliability without compromising participant compliance. Accelerometry raw data was processed with the R-package GGIR [33, 34]. Sustained inactivity and sleep windows were determined using the van Hees 2015 algorithm [35]. Non-sleep time was categorized into inactivity (acceleration threshold, < 25 milligravity [m*g*] of Euclidian Norm Minus One, 1 mg = 0.00981 m.s^−2^), light-intensity (25- < 100 mg), moderate-intensity (100- < 430 mg), and vigorous-intensity physical activity (≥ 430 mg) using prediction equations provided by Hildebrand et al. [36, 37] While inactivity resembles sedentary behavior, it is important to note that wrist-worn accelerometers cannot detect posture, which is a key aspect of defining sedentary behavior. Therefore, inactivity serves as a proxy for sedentary behaviors [37, 38], and the term “sedentary behavior” is consistently used throughout this article. To cover the entire 24-h movement spectrum, overall (non‐bouted) physical activity was the main outcome in the descriptive analyses. Additionally, to evaluate the impact of bout criteria on MVPA estimates, we conducted a supplementary analysis using a 1-min bout definition, where at least 80% of the time within each minute had to meet the 100 mg threshold criteria for MVPA [39].

### Baseline sociodemographic and clinical characteristics

Baseline sociodemographic and clinical characteristics were collected at the first preconception clinic visit using an enrollment questionnaire. Data presented included age, ethnicity (Chinese, Malay, Indian, or mix/none of the above), highest education level (post-secondary and below, university degree, or professional/higher degree), employment status (unemployed or employed), and parity (nulliparous or primiparous/multiparous). Height was measured to the nearest 0.1 cm using a SECA 213 portable rangefinder, and weight was measured to the nearest 0.1 kg using a SECA 803 weighing machine. Body mass index (BMI) was calculated as weight (kg) divided by height (m^2^) and categorized using Asian-specific thresholds (underweight: < 18.5 kg/m^2^, normal weight: 18.5–22.9 kg/m^2^, overweight: 23.0–27.4 kg/m^2^, obese: ≥ 27.5 kg/m^2^). Underweight participants were grouped with normal weight in the analysis.

### Statistical analysis

Statistical analyses were conducted using R version 4.3.1 (R Foundation for Statistical Computing, Vienna, Austria). Accelerometer-measured data were included if women had measurements across all three timepoints or at least two consecutive timepoints: 1) preconception and 24–28 weeks of pregnancy, or 2) 24–28 weeks pregnancy and 12 months postpartum. The assumption of data missing completely at random (MCAR) was assessed by comparing baseline characteristics of participants who contributed complete data to those with only preconception and mid-pregnancy data or only mid-pregnancy and postpartum data (as detailed in the Results section). Differences in continuous variables were evaluated using t-tests, while categorical variables were analyzed using chi-square tests or Fisher’s exact tests when expected cell counts were below five. Given that the assumption of MCAR appeared reasonable after this assessment, Generalized Estimating Equations (GEE) were used for the primary analysis to assess changes in movement behaviors across timepoints.

The three timepoints at preconception, mid-pregnancy, and postpartum were treated as repeated measures and coded as an ordinal variable (0, 1, and 2) using an unstructured correlation structure. The means and 95% confidence intervals (CI) of the variables were then reported for each timepoint. To assess whether there were differences in movement behaviors across timepoints, pairwise t-tests with a Bonferroni correction were conducted between timepoints 1 and 2, 1 and 3, and 2 and 3 after fitting the GEE model. A Wald test was performed for a global assessment of differences in movement behaviors across the entire study duration. Differences in movement behaviors between weekdays and weekends were assessed using pairwise t-tests with a Bonferroni correction for each timepoint separately by fitting the GEE model. Since our analysis focused on descriptive statistics to describe overall trends and changes across timepoints within the constant sample, adjustments for demographic variables were not included.

## Results

**Fig. 1:**
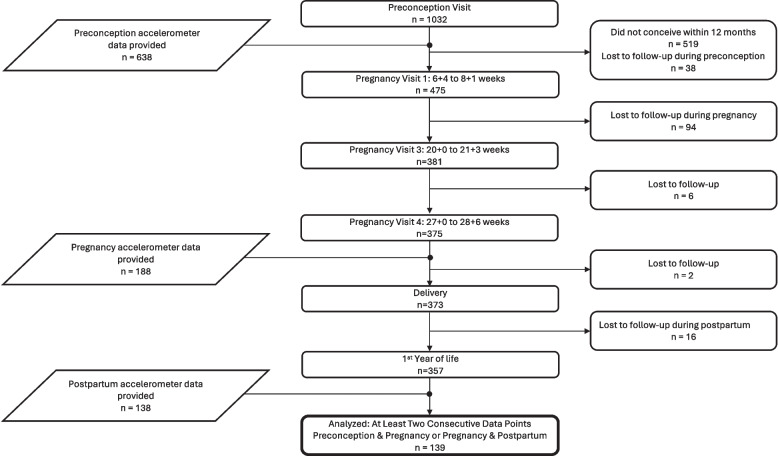
Participants flow chart

The analysis included 139 women who provided data across either all three timepoints or for at least two consecutive time points (i.e., preconception-pregnancy or pregnancy-postpartum) and met the criteria of ≥ 16 h/day of data on at least two weekdays and one weekend day (Fig. 1). Participant baseline characteristics are presented in Table 1. The mean age was 30.8 years. Most women were underweight or of normal weight (61.9%), including 6.5% who were underweight and 55.4% who were of normal weight. The majority identified as Chinese (83.5%), held an undergraduate degree as their highest level of education (59.0%), were employed (88.5%), and were nulliparous (65.5%). Participants wore the accelerometer for an average of 7.4 (standard deviation [SD] 1.4), 7.5 (2.3), and 7.3 (1.2) valid days during preconception, mid-pregnancy, and postpartum, respectively (Table 2). Total waking wear time was consistent across timepoints, averaging 16.9 (SD 0.8), 16.8 (1.0), and 17.2 (1.0) hours/day. Supplementary Table 1 presents the assessment of whether data at different timepoints were MCAR. No differences were observed in all baseline characteristic—age, BMI, ethnicity, education level, employment status, or parity—between groups (all *p* > 0.05), supporting the MCAR assumption and justifying the use of GEE for our analysis.

**Table 1:** Baseline characteristics of women included in the accelerometry study within the S-PRESTO cohort

Baseline characteristics	*n* = 139
Age (mean [SD], years)	30.8 ± 3.5
BMI, Asian cut-offs (n, %)	
Under & normal weight (< 23 kg/m^2^)	86 (61.9)
Overweight (23–27.4 kg/m^2^)	31 (22.3)
Obese (≥ 27.5 kg/m^2^)	21 (15.1)
Missing	1 (0.7)
Ethnicity (n, %)	
Chinese	116 (83.5)
Malay	14 (10.1)
Indian	6 (4.3)
Mix/None of the above	3 (2.2)
Highest level of education (n, %)	
Post-secondary and below	35 (25.2)
University degree	82 (59.0)
Professional/Higher degree	22 (15.8)
Missing	0
Employment status (n, %)	
Unemployed	16 (11.5)
Employed	123 (88.5)
Missing	0
Parity (n, %)	
Nulliparous	91 (65.5)
Primiparous/Multiparous	48 (34.5)

*BMI* body mass index, *SD* standard deviation

**Table 2:** Valid wear days and total waking wear time across preconception, pregnancy, and postpartum

	**Preconception**^**a**^	**Pregnancy**	**Postpartum**
Valid wear days, mean (SD)	7.4 (1.4)	7.5 (2.3)	7.3 (1.2)
Total waking wear time, hours/day	16.9 (0.8)	16.8 (1.0)	17.2 (1.0)

*SD* standard deviation

^a^Only those who eventually became pregnant were included

**Fig. 2:**
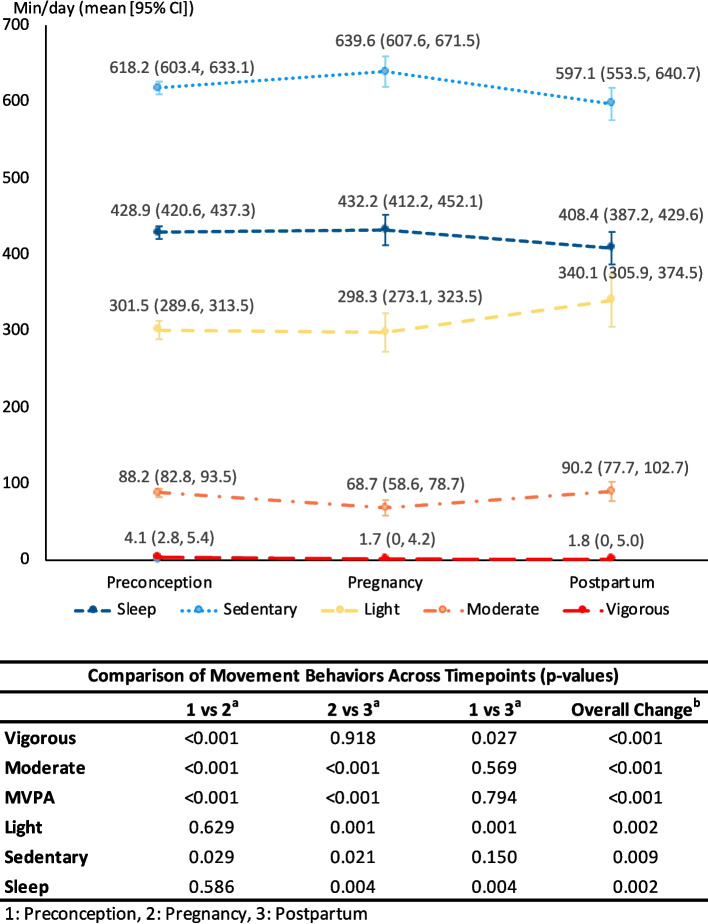
Means and 95% CI for 24-h movement behaviors (min/day) across preconception, pregnancy, and postpartum (*n* = 139), and comparison across timepoints Mean and 95% CI were estimated using Generalized Estimating Equations (GEE). ^a^Pairwise comparisons between timepoints were conducted using t-tests with Bonferroni correction. ^b^Overall change in means across all three timepoints was assessed using the Wald test. Note: MVPA (moderate-to-vigorous physical activity) is not presented in the figure, as it represents a combined measure of both moderate and vigorous intensity physical activity. Abbreviations: CI; confidence interval, MVPA; moderate-to-vigorous intensity physical activity

Overall, there were changes in all movement behaviors across the three timepoints (all *p* < 0.01, Fig. 2). Vigorous-intensity activity dropped from preconception (mean [95% CI]: 4.1 [2.8–5.4)] min/day) to mid-pregnancy (1.7 [0–4.2] min/day; *p* < 0.001 for preconception vs. mid-pregnancy) and remained low postpartum (1.8 [0–5.0] min/day, *p* = 0.918 vs. mid-pregnancy). Moderate-intensity activity also decreased from preconception (88.2 [82.8–93.5] min/day) to mid-pregnancy (68.7 [58.6–78.7] min/day; *p* < 0.001) but rebounded postpartum (90.2 [77.7–102.7] min/day; *p* < 0.001 vs. mid-pregnancy). Light-intensity activity remained stable from preconception (301.5 [289.6–313.5] min/day) to mid-pregnancy (298.3 [273.1–323.5] min/day, *p* = 0.629) but increased postpartum (340.1 [305.9–374.5] min/day; *p* = 0.001 vs. mid-pregnancy), exceeding preconception levels. Sedentary behavior increased from preconception (618.2 [603.4–633.1] min/day) to mid-pregnancy (639.6 [607.6–671.5] min/day; *p* = 0.029) and decreased postpartum (597.1 [553.5–640.7] min/day; *p* = 0.021 vs. mid-pregnancy), returning to preconception levels. Sleep duration remained stable from preconception (428.9 [420.6–437.3] min/day) to mid-pregnancy (432.2 [412.2–452.1] min/day; *p* = 0.586]) but decreased postpartum (408.4 [387.2–429.6] min/day; *p* = 0.004 vs. mid-pregnancy), falling below preconception levels.

MVPA, a combination of vigorous- and moderate-intensity activity, though predominantly moderate, declined from preconception (91.9 [86.2–97.6] min/day) to mid-pregnancy (70.4 [60.0–80.9] min/day; *p* < 0.001 for preconception vs. mid-pregnancy) but rebounded postpartum (92.9 [79.8–105.9] min/day; *p* < 0.001 vs. mid-pregnancy), returning to preconception levels. When applying the 1-min bout definition (requiring 80% of each minute to meet the MVPA threshold), MVPA followed a similar trend but at lower absolute values: 33.3 (30.1–36.5), 21.9 (16.0–27.8), and 26.5 (19.6–33.3) min/day during preconception, mid-pregnancy and postpartum, respectively (Supplementary Fig. 1).

**Fig. 3:**
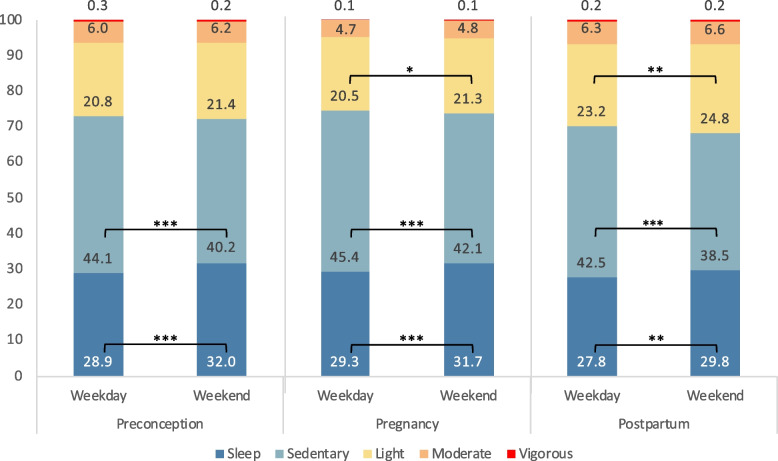
Percentage distribution of wear time across 24-h movement behaviors during preconception, pregnancy, and postpartum by weekdays and weekends (*n* = 139). * *p* < 0.05, ** *p* < 0.01, *** *p* < 0.001 denote statistical differences in 24-h movement behavior between weekdays and weekends across preconception, pregnancy, and postpartum

The daily distribution of 24-h movement behaviors analyzed by weekdays and weekends during each timepoint is illustrated in Fig. 3. No weekday-weekend differences in vigorous-intensity activity were observed (*p* > 0.05); percentages ranged from 0.1% to 0.3% of wear time. Although there was a general trend toward more moderate-intensity activity on weekends, the percentage distributions across weekdays and weekends were comparable at all three timepoints (preconception, mid-pregnancy, and postpartum) (*p* > 0.05), ranging from 4.7% to 6.6% of wear time. During both mid-pregnancy and postpartum phases, women spent more time engaging in light-intensity activity on weekends (21.3%–24.8%) compared to weekdays (20.5%–23.2%; *p* < 0.05). Across all three timepoints, women exhibited higher sedentary behavior during weekdays (42.5%–45.4%) compared to weekends (38.5%–42.1%; *p* < 0.001), while allocating more time to sleep during weekends (29.8%–32.0%) compared to weekdays (27.8%–29.3%; *p* < 0.01).

## Discussion

This longitudinal study prospectively examined accelerometer-measured 24-h movement behaviors across preconception, mid-pregnancy, and 12-months postpartum in a multi-ethnic cohort in Singapore. Our findings partially supported the hypotheses: from preconception to mid-pregnancy, vigorous- and moderate-intensity activities decreased; however, in the postpartum period, while vigorous-intensity activity remained low, moderate-intensity activity rebounded, contrary to our expectation of persistent declines in both. Light-intensity activity remained unchanged from preconception to mid-pregnancy, as hypothesized, and increased postpartum. Sedentary behavior increased during mid-pregnancy and declined postpartum to preconception levels, aligning with our hypotheses. Sleep duration declined during pregnancy and further reduced postpartum, falling below preconception levels as predicted. For weekday-weekend differences, vigorous- and moderate-intensity activities unexpectedly showed no variation. While weekdays were associated with less light-intensity activity during mid-pregnancy and postpartum, weekends showed lower sedentary behavior and longer sleep duration across all timepoints, consistent with our hypotheses.

Few studies have examined physical activity changes from preconception to postpartum and early motherhood [15, 22–24], with most relying on self-reports. For example, a longitudinal study by Hesketh et al. [15] reported accelerometry data but only for early motherhood (4–7 years postpartum), with before/during pregnancy data being self-reported. Our accelerometer data revealed minimal vigorous-intensity activity at preconception (1.7 min/day) that persisted throughout mid-pregnancy and postpartum. This decline, though of uncertain clinical significance, represents a meaningful behavioral shift. Aligning with our findings, da Silva et al. [40] reported similarly low vigorous-intensity activity (3 min/day using a non-bouted criterion, and 1 min/day using a bout criterion of ≥ 1 min with 80% MVPA epochs). An earlier study from the National Health and Nutrition Examination Survey (NHANES) using waist-worn ActiGraph accelerometers reported even lower estimates of vigorous-intensity activity, with values ranging from 0.2–0.4 min/day (Troiano cut-points) and 0.3–0.7 min/day (Swartz cut-points) across trimesters [41]. These differences in estimates likely reflect the influence of methodological choices on activity quantification. Nevertheless, a previous review found that pregnant women who engaged in vigorous exercise 2–3 times per week were able to improve or maintain fitness without affecting pregnancy duration [42], highlighting a potential gap between recommendations (i.e., to maintain preconception activity levels) [6] and the observed decline during pregnancy. This decline may reflect concerns about physical discomfort, safety, or fetal impact [43, 44].

We observed a 19.5 min/day decline in moderate-intensity activity from preconception to mid-pregnancy. Our cohort showed higher MVPA estimates compared to previous studies with accelerometry measurements at two to three pregnancy timepoints –Whitaker et al. [45] (hip-worn ActiGraph): 43–52 min/day; Badon et al. [46]: 35–38 min/day; Sandborg et al. [47]: 18–32 min/day; and Kracht et al. [48]: 13–20 min/day. While wrist-worn monitors may yield higher counts than hip placement [36], our higher MVPA estimates primarily reflect the use of non-bouted processing criteria. Both da Silva et al. [40] and our study applied non-bouted and bouted criteria for comparison. Under non-bouted processing, da Silva et al. [40] reported > 90 min/day of MVPA in the second trimester, exceeding our non-bouted estimates (~ 70 min/day). With the bouted criteria (≥ 1 min with 80% of epochs ≥ MVPA cut-point), da Silva et al. [40] reported ~ 36 min/day of MVPA, which, while slightly higher, is in a comparable range to Kracht et al. [48] and our estimates (~ 22 min/day of bouted MVPA during mid-pregnancy). These results illustrate the impact of activity interruptions on MVPA quantification. Although we did not directly measure activity interruptions, transportation walking (which accounted for most MVPA in an adult population in prior research from Singapore [49]) may exemplify how bout criteria may underestimate MVPA. Brief interruptions (e.g., 30-s to 2-min pauses at traffic lights) could exclude otherwise valid MVPA minutes under an 80% bout threshold, despite participants engaging in predominantly moderate-intensity movement. In the present study, we aimed to report the full spectrum of activity, including sporadic bursts, to capture a more inclusive and ecologically valid representation of overall MVPA, reflecting the inherent variability of free-living activity patterns. Beyond bout criteria, different intensity thresholds also influence MVPA estimates. Compared to our study’s thresholds, newer intensity thresholds by Mielke et al. [50] (e.g., ≥ 25 mg for light, ≥ 78 mg for moderate, ≥ 249 mg for vigorous) may yield higher estimates of physical activity and correspondingly lower sedentary time. These data further demonstrate the influence of methodological choices on accelerometry estimates, which may subsequently affect evaluations of health outcomes and physical activity recommendations.

Notably, while MVPA exceeded the ≥ 150 min/week guideline [6] at all timepoints, this likely reflects methodological differences: the guideline is based on self-reported, bouted activity [51], whereas our accelerometry captured both sustained and sporadic MVPA. Discrepancies between device-measured and self-reported activity are well-documented [52, 53], underscoring the need to refine guidelines to better integrate device-based evidence [51]. Given these differences, we did not directly convert daily MVPA values to weekly totals, maintaining data granularity and avoiding assumptions of consistent activity patterns across days. Despite methodological variations, all previous studies reported declines in physical activity, regardless of intensity, from preconception to pregnancy [15, 22, 23]. During postpartum, while the observed rebound in moderate-intensity activity is encouraging, these levels returned to those observed during preconception. Randomized trials showed that moderate- or vigorous-intensity activity during pregnancy does not harm pregnancy progression or offspring health [54–56]. Interventions promoting awareness and adherence to guidelines are needed to support optimal activity levels throughout pregnancy and beyond.

Light-intensity physical activity remained stable from preconception to mid-pregnancy, only partially offsetting the decline in moderate-intensity activity. This stability, combined with increased mid-pregnancy sedentary behavior, implies replacement of higher-intensity with lower-intensity movement. Our light-intensity activity estimates during pregnancy exceeded those of McParlin et al. [57] (120–122 min/day across trimesters) and Sandborg et al. [47] (198–210 min/day at 14–37 gestational weeks) but were lower than Badon et al. [46] (384–408 min/day, early-late pregnancy). Methodological, temporal, and population differences may explain these variations. Postpartum, light-intensity activity increased, replacing some sedentary time and aligning with WHO recommendations for health [6]. This rise mirrored Kracht et al. [48] (~ 160 min/day during pregnancy; 196 min/day postpartum).

In this study, sedentary behavior increased by an average of 21.4 min/day from preconception to mid-pregnancy. However, at 12 months postpartum, it returned to near preconception levels, reflecting a decrease of 42.5 min/day from mid-pregnancy. Compared to our estimates, Badon et al. [46] reported lower sedentary behavior during pregnancy (532–536 min/day), and McParlin et al. [57] observed median sedentary time ranging from 585–631 min/day across trimesters. Whitaker et al. [45] reported higher sedentary time across trimesters: 936 min/day (first), 907 min/day (second), and 912 min/day (third). Kracht et al. [48] found a similar trajectory, with sedentary time increasing from 618 min/day in early pregnancy to 630 min/day in late pregnancy, then decreasing to 588 min/day at 1 year postpartum, closely aligning with our findings. These variations, along with the generally higher sedentary time observed in device-based studies compared to self-reported measures [15, 23], highlight the methodological and population-level differences influencing sedentary behavior estimates.

Accelerometer-measured sleep duration in our study remained stable from preconception to mid-pregnancy, contrasting with previous research reporting declines in self-reported sleep duration beginning in the second trimester [58, 59]. This discrepancy may stem from retrospective recall method of data collection [58]. Our assessment period, which occurred during the second trimester, captured a time when sleep quality begins to decline but may not yet reach the levels of disruption as seen more commonly in the third trimester [60]. A meta-analysis [61] of 16 actigraphy studies, most of which assessed the third trimester, found a 10.8% decline in sleep duration from pregnancy to 6 months postpartum. In contrast, our study observed a smaller reduction of 5.5% in sleep duration (equivalent to 23.8 min/day) from mid-pregnancy, which persisted up to 12 months postpartum. Mothers in our study averaged 7.2 h/day of sleep during mid-pregnancy, but this decreased to 6.8 h/day postpartum—4.8% (20.5 min/day) lower than preconception levels. Notably, this 4.8% reduction at 12 months postpartum may underestimate sleep disruption earlier in the postpartum period, as infant sleep schedules are often less established in the first few months [62]. Future research may include assessments across the postpartum period (e.g., 3, 6, and 9 months) to better capture dynamic changes in sleep and activity patterns. Interventions addressing maternal sleep are crucial to prevent worsening sleep problems over time.

While no significant weekday-weekend differences were found for vigorous- or moderate-intensity activity at any timepoint, there was an overall trend of increased physical activities on weekends. Light-intensity activity levels were higher on weekends compared to weekdays during mid-pregnancy and postpartum. On weekends, sedentary behavior was consistently lower across all timepoints, while sleep duration was consistently higher. These patterns suggest weekends favor more light-intensity activity and longer sleep, whereas weekdays are marked by higher sedentary behavior. To our knowledge, no studies have specifically examined weekday-weekend differences in 24-h movement behaviors among mothers for direct comparison. Nonetheless, several device-based studies on parent–child behaviors have been identified, reporting mixed findings: some found higher physical activity on weekends [25], while others reported lower activity, [26] reduced step counts [27], or increased sedentary behavior [26]. These inconsistencies highlight the need for further research on weekday-weekend differences in maternal movement behaviors across the preconception-to-postpartum transition—an area that remains underexplored. Future research could explore the specific activities driving these patterns and their potential implications for maternal and offspring health. Interventions might focus on promoting structured activities (e.g., active commuting) during weekdays and encouraging family-based activities (e.g., park visits) on weekends to support healthier movement behaviors [63].

### Strengths and limitations

Strengths of our study include its longitudinal, prospective analysis of maternal 24-h movement behaviors, capturing temporal variations across preconception, mid-pregnancy, and postpartum. The exploration of weekday-weekend variations added nuance by considering potential lifestyle differences, while accelerometry enhanced reliability and minimized social desirability bias inherent in self-reports. Additionally, our study is among the first to use accelerometry to prospectively track 24-h movement behaviors from preconception, providing a comprehensive understanding of their evolution across key reproductive stages.

Some limitations should be noted. Our study population may not represent the broader Singaporean female population, particularly those who have not experienced pregnancy, limiting generalizability. While alternative processing methods, such as those proposed by Mielke et al. [50], would yield different absolute estimates of activity volumes, the temporal trends (e.g. the observed decline in moderate-intensity activity from preconception to pregnancy) we observed would likely remain consistent. Nevertheless, our findings should be interpreted in the context of the specific intensity cut-points and bout criteria employed. Accelerometry data did not differentiate between leisure and occupational physical activity, which may have distinct health implications [64–66], a limitation common in accelerometry studies. While data collection over seven consecutive days is widely employed, the dynamic changes across the entirety of the preconception, pregnancy, and postpartum periods were not fully captured. Requiring only one weekend day for valid accelerometry data may limit the representativeness of weekend movement patterns. The study also did not capture factors influencing weekend activity patterns, such as work schedules or childcare responsibilities. The lack of sleep diaries or non-wear logs may have led to misclassification of sleep and wake periods, potentially impacting the accuracy of accelerometer-derived sleep estimates. However, given that the accelerometer was wrist-worn, it is unlikely participants remained completely still for > 15 min during wakefulness, as even sedentary activities (e.g., sitting, working) typically involve intermittent wrist movements. Wrist-worn accelerometers are also well-validated for detecting sleep–wake patterns [35].

Future research should assess adherence to 24-h movement profiles (currently adult-only) for pregnancy monitoring and develop pregnancy-specific 24-h movement guidelines, integrating physical activity, sedentary behavior, and sleep quality (beyond duration) to optimize maternal and fetal health. The variability in processing criteria highlights the need for continued development of standardized accelerometry thresholds and device-based physical activity recommendations to improve classification consistency, facilitate cross-study comparisons, and advance our understanding of physical activity patterns. Incorporating sleep logs, assessing movement behaviors across all trimesters, and exploring paid leave policies as a covariate could provide a more comprehensive understanding of activity patterns during pregnancy and postpartum. Interventions should address barriers such as physical discomfort, childcare responsibilities, and work schedules, while policymakers could promote workplace flexibility and paid leave to support maternal health. These efforts would address current guideline limitations and enhance the relevance of 24-h movement behaviors research.

## Conclusions

During pregnancy, vigorous- and moderate-intensity activity declined, with vigorous-intensity activity remaining low postpartum. Light-intensity activity increased postpartum, while sedentary behavior increased during pregnancy. Sleep duration decreased postpartum. Vigorous- and moderate-intensity activity showed no weekday-weekend differences. Weekdays were marked by less light-intensity activity during pregnancy and postpartum and higher sedentary behavior across all timepoints, while weekends had more sleep across timepoints. These findings highlight key targets for improving maternal health: promoting vigorous- and moderate-intensity activity during pregnancy and beyond, reducing sedentary time on weekdays, and supporting light-intensity activity and sleep, particularly on weekdays.

## Supplementary Information

Supplementary Material 1.

## Data Availability

The dataset supporting the conclusions of this article can be made available upon request and after approval by the S-PRESTO Executive Committee.
