# Supplementary material for: Preconception to postpartum accelerometry-based 24-hour movement behaviors: a prospective cohort study
Source: BMC Public Health. 2026 Jan 7;26:465. doi: 10.1186/s12889-025-26034-4 (PMC12870911; doi:10.1186/s12889-025-26034-4)
Supplement: Supplementary file 1 — Supplementary Material 1. [file 12889_2025_26034_MOESM1_ESM.docx]

**Title:** Preconception to Postpartum Accelerometry-based 24-Hour Movement Behaviors: A Prospective Cohort Study

**First Author**: Anne HY Chu

Table of Contents

[Supplementary Table 1. Comparison of baseline characteristics of participants who contributed complete data versus those with only timepoints 1-2 or 2-3. 2](#_Toc194587456)

[Supplementary Figure 2. MVPA means (95% confidence intervals) across preconception, pregnancy, and postpartum by bout criteria. 3](#_Toc194587457)

# Supplementary Table 1. Comparison of baseline characteristics of participants who contributed complete data versus those with only timepoints 1-2 or 2-3.

| **Baseline characteristics** | **Group 1 (complete)** | **Group 2^a^  (T1-T2)^b^** | **Group 3^a^  (T2-T3)^b^** | **Group 1 vs. Group 2** | **Group 1 vs. Group 3** |
| --- | --- | --- | --- | --- | --- |
|  | **n=62** | **n=69** | **n=23** | **p-value^c^** | **p-value^c^** |
| **Age [mean (SD)]** | 30.9 (3.5) | 30.7 (3.7) | 30.6 (2.8) | 0.760 | 0.691 |
| **BMI, Asian cut-offs (n, %)** |  |  |  | 0.908 | 0.192 |
| Under & normal weight (<23 kg/m²) | 38 (61.3%) | 43 (63.2%) | 11 (47.8%) |  |  |
| Overweight (23-27.4 kg/m²) | 12 (19.4%) | 14 (20.6%) | 9 (39.1%) |  |  |
| Obese (≥27.5 kg/m²) | 12 (19.4%) | 11 (16.2%) | 3 (13%) |  |  |
| **Ethnicity (n, %)** |  |  |  | 0.784 | 0.213 |
| Chinese | 49 (79%) | 51 (75%) | 21 (91.3%) |  |  |
| Malay | 8 (12.9%) | 11 (16.2%) | 0 (0%) |  |  |
| Indian | 3 (4.8%) | 5 (7.4%) | 2 (8.7%) |  |  |
| Mix/None of the above | 2 (3.2%) | 1 (1.5%) | 0 (0%) |  |  |
| **Highest level of education (n, %)** |  |  |  | 0.791 | 0.427 |
| Post-secondary and below | 17 (27.4%) | 21 (30.4%) | 4 (17.4%) |  |  |
| University degree | 36 (58.1%) | 35 (50.7%) | 16 (69.6%) |  |  |
| Professional/Higher degree | 9 (14.5%) | 13 (18.8%) | 3 (13%) |  |  |
| **Employment status (n, %)** |  |  |  | 0.567 | 0.666 |
| Unemployed | 6 (10.9%) | 7 (14.9%) | 1 (4.8%) |  |  |
| Employed | 49 (89.1%) | 40 (85.1%) | 20 (95.2%) |  |  |
| **Parity (n, %)** |  |  |  | 0.819 | 0.752 |
| Nulliparous | 39 (62.9%) | 41 (59.4%) | 16 (69.6%) |  |  |
| Primiparous/Multiparous | 23 (37.1% | 28 (40.6%) | 7 (30.4%) |  |  |

^a^ Group 2 explicitly excludes observations where T3 data is available. Group 3 explicitly excludes observations where T1 data is available.

^b^ T1: preconception, T2: pregnancy, T3: postpartum.

^c^ A t-test was used to assess differences in continuous baseline characteristics between groups, while categorical variables were compared using a chi-square test or Fisher’s exact test when expected cell counts were below five.

Note: Discrepancies in totals are due to missing values.

Abbreviation: BMI, body mass index

# Supplementary Figure 2. MVPA means (95% confidence intervals) across preconception, pregnancy, and postpartum by bout criteria.

Abbreviation: MVPA, moderate‐to‐vigorous physical activity
